# Successful Young Athletes Have Low Probability of Being Ranked Among the Best Senior Athletes, but This Is Higher When Compared to Their Less Successful Peers

**DOI:** 10.3389/fpsyg.2022.869637

**Published:** 2022-06-02

**Authors:** Eduard Bezuglov, Anton Emanov, Zbigniew Waśkiewicz, Nadezhda Semeniuk, Mikhail Butovsky, Maria Shoshorina, Daria Baranova, Kristina Volodina, Ryland Morgans

**Affiliations:** ^1^Department of Sports Medicine and Medical Rehabilitation, Sechenov First Moscow State Medical University of the Ministry of Health of the Russia Federation, Moscow, Russia; ^2^High Performance Sport Laboratory, Moscow Witte University, Moscow, Russia; ^3^Sirius University of Science and Technology, Sochi, Russia; ^4^Professional Football Club CSKA, Moscow, Russia; ^5^“Smart Recovery” Sports Medicine Clinic LLC, Moscow, Russia; ^6^Institute of Sport Science, Jerzy Kukuczka Academy of Physical Education, Katowice, Poland; ^7^Football Club “Rubin”, Kazan, Russia

**Keywords:** talent, track and field, career trajectory, sporting success, young athletes, senior athletes

## Abstract

**Background:**

Contemporary research has shown that only a small proportion of high achieving young athletes continue to become successful senior athletes. However, there is a lack of empirical literature tracking the success of senior male and female athletes who were considered high achieving as a youth.

**Hypothesis:**

Athletes of both sexes who are successful in youth categories (U18 and U20) are more likely to be successful senior athletes.

**Conclusion:**

Data from 67,600 athletes were collated from the tilastopaja.org platform. The inclusion criteria for both genders were determined by top-100 ranking in the U18 and U20 age groups and progression to the top-100 as a senior athlete. Only 23.5% of successful track and field athletes (ranked in top-100) at U18 became a successful senior athlete, while 35.4% were from the U20 group. Girls ranked in the top-100 U18 and U20 categories are significantly more likely to be ranked in the top-100 as a senior when compared to boys. Although, being ranked in the top-50 at U18 and U20 significantly increases the probability of becoming a successful senior athlete when compared with less successful athletes at these age groups (*p* < 0.001). Notably, the majority (68.5%) of the most successful senior athletes were not ranked in the top-100 when in the U18 or U20 age groups. Only a small group of track and field athletes that are successful at U18 and U20 become successful at senior level. The most successful track and field youth athletes are significantly more likely to succeed as a senior athlete than their less successful peers, while girls are more likely to be successful than boys.

## Introduction

Modern professional sports have become more competitive over recent years, mainly due to an increase in sports popularity and a significant rise in financial influence (Till et al., 2019). Therefore, in order to achieve success, sporting organizations may need to create an elite infrastructure and coaching environment, for athletes to develop the physical and psychological qualities required. The development of psychological skills such as coping strategies and the athletes’ ability to assess their own actions and develop identified weaknesses can potentially provide an advantage for young athletes (Saward et al., 2020). However, despite the significance of certain psychological skills (e.g., confidence) and the continuing development, there are still scant literature addressing the psychological and environmental issues that influence talent identification and development (Sarmento et al., 2018; Greenlees et al., 2021).

A key indicator that characterizes leading sports organizations is the percentage of elite young athletes that are successful in adulthood. Although, in the vast majority of cases, only a small percentage of young athletes are successful at senior level (Suppiah et al., 2015). Notably in the literature, there is no accepted definition of talent, which prevents the prediction, with any accuracy, of success in senior sports (Till and Baker, 2020). Currently, the definition of talent during initial sporting academy selection is most often based on the subjective opinion of coaches and trainers, as well as the objective assessment of one or more physical qualities (strength, speed, power, coordination) (Schorer et al., 2017; Roberts et al., 2019, 2021). Due to this approach, it can be difficult to predict adult success in speed-strength sports that specialize early (football, ice hockey, athletics), and in sports where the relative age effect (RAE) and different rates of biological maturation (pre- and post-puberty) are associated (Lovell et al., 2015; Brazo-Sayavera et al., 2017; Sierra-Díaz et al., 2017). These factors can also lead to selection process bias and exclusion from highly competitive sports for the “late-maturing” athlete (Dugdale et al., 2021).

Several authors have previously highlighted the significance of developing physical and mental qualities in biologically maturing young athletes of both sexes aged 11–16 years (Baker et al., 2014; Gouvea et al., 2016; Emmonds et al., 2020; Itoh and Hirose, 2020). Furthermore, Romann and Cobley (2015) found that a 1-year age difference resulted in average expected performance differences of 5.3–10.1% in 8–15 year-old athletes. Fundamentally, relative age and maturation operate independently and reducing their influence on selection bias should occur (Hill et al., 2020a). Leading sports organizations are aware of these current limitations and have attempted to develop measures to reduce the possible negative influence during assessment (Hill et al., 2020b; Helsen et al., 2021; Lüdin et al., 2021). For example, in football, “bio-banding” has been implemented to reduce bias associated with different stages of biological maturation, and has been proven to be highly effective in elite young athletes (Hill et al., 2020b; Helsen et al., 2021; Lüdin et al., 2021). The concept is designed to group young athletes based on maturity status rather than chronological age (Malina et al., 2019). Maturation status has been shown previously to affect a range of performance and anthropometric parameters in young football players, including technical performance during small-sided games, accumulated training load, and aerobic and speed performance during the competitive season (Moreira et al., 2017; Nobari et al., 2021a,b; Eskandarifard et al., 2022). Furthermore, separating athletes by maturity status (most commonly peak height velocity or percentage of estimated adult stature) may reduce overuse injuries in young athletes at different stages of maturation and thus promote continued participation in sport (Towlson et al., 2021).

In contrast, all major young athletic competitions are grouped according to chronological age, which is likely to create the over-representation of early-born athletes, especially in 16–18 year old male athletes (Kearney et al., 2018; Brustio et al., 2019). Numerous studies have supported this notion, particularly in football and athletics where the dominance of early-born athletes over late-born athletes in elite competitions and international tournaments is evident (Hollings et al., 2014a; Brustio et al., 2019; De la Rubia et al., 2020; Doncaster et al., 2020; Brustio and Boccia, 2021; Figueiredo et al., 2021). However, it has also been reported that at an elite senior level, the number of late-born athletes is much greater although some authors documented a RAE reverse in the most competitive athletes (Fumarco et al., 2017; Bezuglov et al., 2020). Late-born athletes are not regarded as less talented but their under-representation in elite youth sport has been associated with physical and hormonal profiling, the development of which is directly related to the stage of maturation (Murtagh et al., 2018; Almeida-Neto et al., 2020, 2021).

This problem not only exists in highly competitive team sports (hockey, football, baseball), but also in athletics (Brazo-Sayavera et al., 2018; Kearney et al., 2018). This concept has been purported by several studies that examined the career trajectory of young athletes from various disciplines (Boccia et al., 2019). Boccia et al. (2019) found that a later start in competitive sports and a career past the age of 23–25 years was an important factor in achieving better results in sprinters and throwers. Therefore, several conclusions can be drawn regarding the possible factors of low transition from elite youth to elite senior sport. Notably, the potential damaging effect of in-appropriate physical training of successful young athletes, which can lead to functional over-reaching, increased injuries and drop-out from highly competitive professional sports. It can therefore be assumed that successful young athletes are more likely to remain successful as adults when compared to their less successful peers. However, a comparative assessment of the career trajectory of successful young athletes to senior athletes, has not yet been conducted. The findings of such research would be of great practical interest. Therefore, the aim of this study was to investigate the success of senior athletes of both sexes who were an elite performer in their youth.

## Materials and Methods

### Participants

The results of all male and female athletes who were ranked on at least one occasion in the top-100 during the period from 1999 to 2020 were analyzed in the following age groups: under 18 (U18), under 20 (U20), and over 20 (senior).

### Experimental Approach to the Problem

According to Hollings et al. (2014b) the age of peak sporting success in male athletes was 23.9 ± 2.4 years (10,000 m) and 28.5 ± 2.2 years (discus throw), and for female athletes was 24.7 ± 2.5 years (pole vault) and 28.1 ± 3.9 years (discus throw). In support of this finding, we concluded that modern athletes reach peak success in the age range 23–28 years, depending on the discipline. Our analysis of U18 athletes was conducted in the period 1999–2010, U20 in the period 1999–2012, and in 1999–2020 for senior athletes. This ensured the maximum number of successful U18–U20 athletes were analyzed between 2010 and 2012, respectively.

Athletes were considered successful in the comparison of age groups in different disciplines. For example, a U18 athlete could be in the top-100 in the hurdles and a U20 athlete could be in the 200 m sprint or all-around.

The site tilastopaja.org was used to collate all data which contained information relating to recorded results at all major athletic competitions. Data from this site has been previously validated (Hamlin et al., 2015; Iljukov and Schumacher, 2017; Montagna and Hopker, 2018). Due to the open-data source employed for data collection, local Ethics Committee approval was not required.

In the primary analysis, data was obtained on all athletes who ranked at least once in the top-100 at U18, U20, and senior categories during the specified study period for these age groups. This first analysis examined the number of athletes ranked in the top-100 (at least once) for both genders at U18 and U20 and who were also ranked in the top-100 (at least once) as a senior, and the career trajectory of the best U18 athletes.

### Procedures Performed

The sample was sub-divided into two groups:

- group one: minimum of one occasion, athletes are ranked in the top-50 at U18 and U20 categories – “successful”;

- group two: minimum of one occasion, athletes are ranked in the top-51–100 but never ranked in the top-50 at U18 and U20 categories – “less successful”;

The number of athletes in the two groups (U18 and U20), ranked in the top-50 as a senior and their career trajectory were analyzed (U18, U20, seniors). These parameters were examined separately for male and female athletes. Additionally, a further examination into the success of male and female athletes at senior level in group one and two was also conducted, including male and females separately and the career trajectory of all athletes in both groups (one and two).

### Statistical Analysis

All statistical analyses were performed using R statistical software version 4.0.5. Wald Chi-square (χ^2^) test was used to evaluate the differences between groups. Odds ratio (OR) and 95% confidence interval (CI) represented U18 and U20 athletes from “successful” and “less successful” groups to be ranked in the top-50 and top-100 as senior athletes. The *p*-value (>0.05) indicated significant differences.

## Results

**FIGURE 1:**
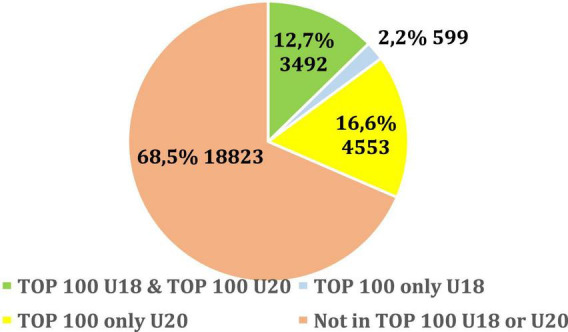
Performance of U18 and U20 athletes who were ranked in the top-100 as seniors.

From 17,435 U18 athletes ranked in the top-100, 4,091 athletes (23.5%) entered the top-100 as a senior athlete. While out of the 22,698 U20 athletes ranked in the top-100, 8,045 athletes (35.4%) were already successful as a senior. Thus, U20 athletes ranked in the top-100 were much more likely to be ranked in the top-100 as a senior when compared with U18 athletes who were ranked in the top-100 (*p* < 0.001; OR = 0.56; 95% CI = 0.53–0.58). From the 17,435 U18 athletes ranked in the top-100, 8,485 athletes (48.6%) were ranked in the top-100 at U20 and 3,492 athletes (20%) were ranked in the top-100 as a senior. Only 20% of young athletes have been consistently successful at U18, U20, and as a senior. Furthermore, 48% of successful U18 athletes were not successful at U20 or as a senior athlete. When analyzing the ranking of the top-100 senior athletes, evidently the majority (68.5%) of 27,467 athletes were not in the top-100 either at U18 or U20 (Figure 1).

**FIGURE 2:**
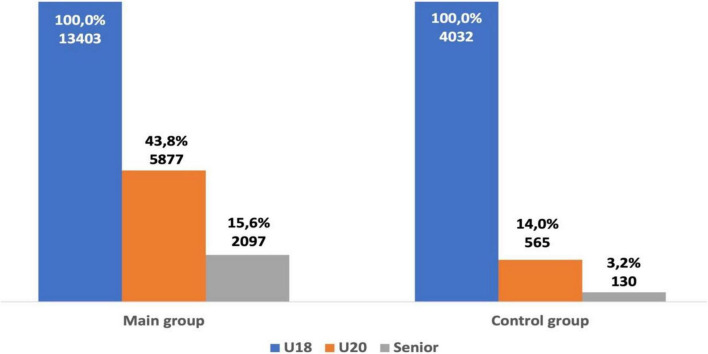
Career trajectory of U18 athletes in group one and group two.

The data from group one and group two were analyzed. From 13,403 U18 athletes in group one, 19.3% (2,586 athletes) of athletes were successful as a senior athlete (top-50). Only 6.7% (272 athletes) of athletes in group two were successful as a senior athlete. U18 athletes from group one were significantly more likely to be successful as a senior athlete when compared with athletes from group two (χ^2^ = 355.19, *p* < 0.001; OR = 3.30; 95% CI = 2.90–3.76). From the 14,897 U20 athletes in group one, 30.7% (4,569 athletes) of athletes were successful as a senior athlete, while only 10.7% (836 athletes) of athletes were successful as a senior athlete from group two. U20 athletes from group one were also more likely to be successful as a senior athlete when compared with athletes from group two (χ^2^ = 1122.6, *p* < 0.001; OR = 3.69; 95% CI = 3.40–3.99). From 13,403 U18 athletes in group one, 5,877 athletes (43.8%) were ranked in the top-50 U20 group and 2,097 athletes (15.6%) retained their success (top-50) as a senior athlete. From the 4,032 U18 athletes in group two, only 565 athletes (14%) were ranked in the top-50 in the U20 category and 130 athletes (3.2%) remained successful as a senior athlete (see Figure 2).

**FIGURE 3:**
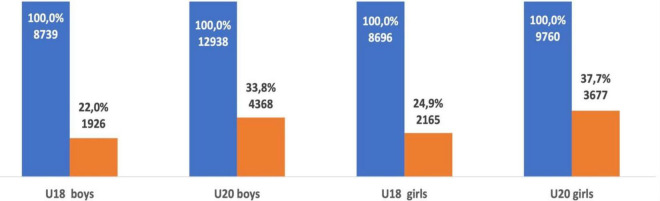
Success of senior male and female athletes from the top-100 at U18 and U20.

When comparing the success of U18 male and female athletes ranked in the top-100, a significant difference was revealed. Female athletes are significantly more likely to be ranked in the top-100 as a senior athlete when compared with male athletes (*p* < 0.01, OR = 0.86; 95% CI = 0.8–0.92). In a similar comparison of U20 successful athletes (male and female), females also retained success as a senior athlete more often than males (*p* < 0.01, OR = 0.8; 95% CI = 0.8–0.89) (see Figure 3).

**FIGURE 4:**
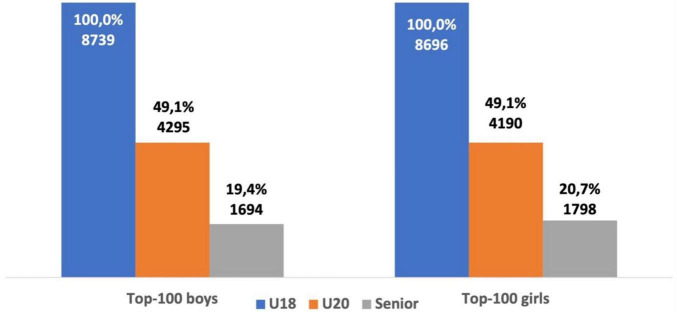
Career trajectory of U18 male and female athletes ranked in the top-100.

When comparing the career trajectories of U18 male and female athletes ranked in the top-100, our data showed that there were no significant differences in the number of athletes consistently successful at U18, U20, and as a senior athlete (see Figure 4).

**FIGURE 5:**
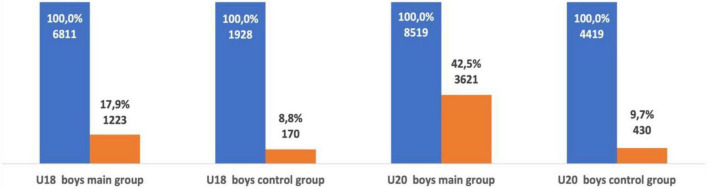
The number of successful U18 and U20 male athletes as a senior in group one and group two.

**FIGURE 6:**
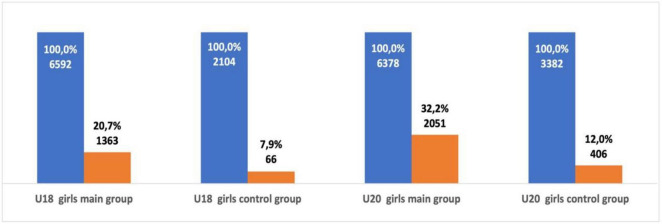
The number of successful U18 and U20 female athletes as a senior in group one and group two.

**FIGURE 7:**
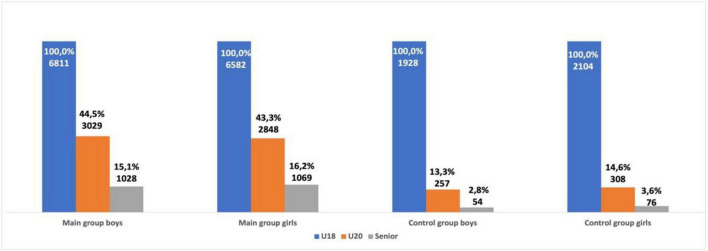
Career trajectory of U18 male and female athletes from group one and group two.

U18 female athletes from group one were ranked significantly more often in the top-50 as a senior athlete when compared with female athletes from group two (χ^2^ = 179.07, *p* < 0.001; OR = 3.04; 95% CI = 2.57–3.60). U20 female athletes from group one were also ranked significantly more often in the top-50 as a senior athlete when compared with athletes from group two (χ^2^ = 475.44, *p* < 0.001; OR = 3.47; 95% CI = 3.09–3.90). U18 male athletes from group one ranked significantly more often in the top-50 as a senior athlete when compared with male athletes from group two (χ^2^ = 179.9, *p* < 0.001; OR = 3.76; 95% CI = 3.06–4.62). U20 male athletes from group one were also more successful as a senior athlete when compared with group two. The probability of U20 male athletes from group one ranking in the top-50 as a senior athlete was significantly higher (χ^2^ = 648.99, *p* < 0.001; OR = 3.89; 95% CI = 3.49–4.34) than group two (see Figures 5, 6). Out of the 6,811 U18 male athletes in group one, 3,029 athletes (44.5%) were ranked in the top-50 at U20 and 1,028 athletes (15.1%) were ranked in the top-50 as a senior athlete. Out of the 1,928 U18 male athletes in group two, only 257 athletes (13.3%) were ranked in the top-50 at U20 and only 54 athletes (2.8%) were successful as a senior athlete. Out of the 6,582 U18 female athletes in group one, 2,848 athletes (43.3%) were ranked in the top-50 at U20 and 1,069 athletes (16.2%) remained successful as a senior athlete. Out of the 2,104 U18 female athletes from group two, 308 athletes (14.6%) were ranked in the top-50 at U20 and 76 athletes (3.6%) remained successful as a senior athlete. Both male and female U18 athletes from group one were more likely to maintain success at U20 and as a senior athlete when compared with group two (see Figure 7).

## Discussion

Our study reported data to suggest that only a small group (23.5% of U18 and 35.4% of U20 athletes) of successful young athletes remain successful as a senior athlete. These findings are consistent with several previous studies conducted in track and field athletes (Piacentini et al., 2014; Boccia et al., 2020, 2021). In a study by Boccia et al. (2020) it was stated that only 17% of U18 male and 21% of U18 female sprinters listed in the top-50 world rankings managed to enter the top-50 rankings as a senior. In another study examining throwers (Boccia et al., 2021), it was found that the frequency of ranking in the top-50 senior athletes who also ranked in the top-50 U16 and U18 age categories was only 6 and 12% for male and 16 and 24% for female athletes, respectively. Furthermore, when assessing jumpers, only 8% of male and 16% of female athletes who were ranked in the top-50 U16 age category managed to be ranked in the top-50 as a senior (Boccia et al., 2021). According to Piacentini et al. (2014) 60% of the most successful throwers competing in the World Junior Championship Finals in 2002 and 2004 were not ranked in the International Athletics Federation after 8–10 years. Similar data were obtained for male and female middle-distance runners who also competed in the World Junior Championship Finals in 2002 and 2004 (Pizzuto et al., 2017). Possible reasons for such a negative transition to elite senior sport may be associated with the characteristics of youth athletics. For example, young athletes who are more physically developed are generally more successful, due to success in most athletic disciplines requiring significant development of speed and strength, however, this may not predict future talent.

Athletics can be characterized by a relatively late start to specialized training where speed and strength are widely regarded as the key qualities that ensure success across all disciplines. Therefore, early-born and early-maturing athletes will initially have an advantage, as they are more likely to be faster and stronger than their younger rivals. In addition, previously successful young athletes are more likely to gain the opportunity to train in better conditions and with the best coaches. Another determining factor in successful youth athletes is the level of testosterone, which significantly correlates with strength and the concentration of testosterone depends on the degree of biological maturation (Cardinale and Stone, 2006). According to Handelsman (2017) prior to puberty, there is no sex difference in circulating testosterone concentrations or athletic performance, while circulating testosterone concentrations in men increase rapidly post-puberty when the testes begin to produce more testosterone (30 times) than pre-puberty. While male blood testosterone concentrations are significantly greater (15 times) than females at any age. In this regard, gender differences in sporting success start to appear at 12–13 years and increase until late adolescence, while a temporary parallel rise to circulating testosterone in boys during puberty is also evident (Handelsman, 2017; Handelsman et al., 2018).

Considering that growth spurts often occur around 13 years old for boys and girls and peak growth is observed at 13–16 years old (Malina et al., 2017), it may be prevalent that in speed-strength competitions for young athletes (10–11 years), categories of two or more years may provide an advantage for the chronologically older athletes with a greater degree of maturation. Thus, it can be assumed that in athletic disciplines where speed and strength are vital qualities, such athletes may be more successful due to early biological maturity. This maturity status provides these athletes with optimal opportunities to produce winning competitive performances due to already part of the best infrastructure and coaching environments, which in turn, further increases the likelihood of success, until the end of growth and maturation (the Matthew effect). However, the progression of athletic improvement and corresponding performances may be slowed after reaching full biological maturity, which can lead to exclusion from highly competitive environments at the senior level, thus highlighting the low transition rate of young athletes to the elite senior level, as evident in our study.

It should also be noted, that a further explanation for the low transition rate of the elite young athlete to the senior level may be the extreme nature of the physical fitness program delivered to the young athletes. This has previously been associated with the coaches and organizations desire to achieve maximum results at major youth international competitions at U16, U18, U20, and U23. In this study, as previously reported in earlier studies (Boccia et al., 2020, 2021) it has been shown that females who were successful as young athletes are more likely than males to achieve success as adults. The most probable physiological reason for this may be the significantly lower rate of testosterone levels in adolescence females and consequently less significance placed on this hormone for sporting success. Another possible reason may be the lack of competition opportunities for female juvenile throwers and senior levels of competition in all athletic disciplines.

The main finding in our study was that less successful young athletes were less likely to succeed as a senior than more successful young athletes. The likelihood of success as a senior athlete is significantly higher in athletes who were more successful at U18 and U20 when compared with athletes who were less successful in the same age groups. Contrastingly, athletes ranked in the top-51–100 were less likely to prepare appropriately for performance at major international competitions and it was deemed un-necessary to enhance their training. Thus, it may be suggested that the low transition rate from youth to senior sport is most likely not related to the extreme nature of the physical fitness plan. Furthermore, successful young athletes, due to an advanced biological status, were able to tolerate long-term high-intensity training loads and may significantly progress in performance and results until the cessation of biological growth. Given that biological maturity status is still not practically applied in athletics, less mature (and probably less successful) young athletes may train inappropriately when following the training programs of their more biologically mature (and successful) peers. This may lead to an increased risk of functional over-reaching, potential trauma, pathological over-exertion and ultimately exclusion from professional sport (Towlson et al., 2021). Therefore, it is the less successful young athletes (chronological age and biological maturity status) who are subjected to over-exertion.

In our study, it was shown for the first time that the likelihood of success as a senior athlete is significantly higher for athletes who were more successful in adolescence compared to their less successful peers. It may be assumed that the small percentage of athletes who were successful both as a young athlete and at the senior level are not the consequence of a “forced” extreme physical fitness program. Additionally, less successful (and probably, less “forced” young athletes) athletes are less likely to progress to an elite senior level. It has also been shown that successful young female athletes are more likely to remain successful at the senior level when compared with young males, which may be due to several factors. Primarily, the increase in females’ testosterone concentration is much less pronounced than in males and this may reduce the positive effect this hormone has on sporting performance and success. Our study also highlighted that 68.5% of elite senior athletes were not successful, that is, they did not rank in the top-100 U18, and did not participate in youth athletic competitions.

Future research should aim to analyze the physical activity of successful senior athletes when in U18 and U20 categories. This will help identify the required level of physical fitness and an appropriate starting age to produce elite senior athletic performance and success. Finally, a further area for consideration may be the development of methods to reduce possible bias when identifying talented young athletes.

## Conclusion

According to the data of our research, only a small group of athletes who are successful at U18 and U20 become successful as a senior athlete. Both male and female athletes who are more successful in young age categories (U18 and U20) were more likely to achieve success as a senior than less successful young athletes. Female athletes who were successful when young were more likely to maintain athletic success as a senior when compared to young males. A significant proportion of senior athletes (both male and female) were not successful in any of the young age categories. Finally, a paradoxical conclusion stating that a later athletic start or engaging in another sport is a more significant predictor of success as a senior athlete compared to sporting success at U18 and U20.

## Data Availability Statement

The original contributions presented in the study are included in the article/supplementary material, further inquiries can be directed to the corresponding author/s.

## Ethics Statement

Ethical review and approval was not required for the study on human participants in accordance with the local legislation and institutional requirements. Written informed consent for participation was not required for this study in accordance with the national legislation and the institutional requirements.

## Author Contributions

EB, ZW, and RM contributed to conception and design of the study. AE, ZW, and NS organized the database. MB and DB performed the statistical analysis. EB and RM wrote the first draft of the manuscript. MS, KV, AE, and MB wrote sections of the manuscript. All authors contributed to manuscript revision, read, and approved the submitted version.

## Conflict of Interest

AE and NS were employed by the “Smart Recovery” Sports Medicine Clinic LLC. EB was employed by company Professional Football Club CSKA. MB was employed by company Football Club “Rubin”, Kazan. The remaining authors declare that the research was conducted in the absence of any commercial or financial relationships that could be construed as a potential conflict of interest.

## Publisher’s Note

All claims expressed in this article are solely those of the authors and do not necessarily represent those of their affiliated organizations, or those of the publisher, the editors and the reviewers. Any product that may be evaluated in this article, or claim that may be made by its manufacturer, is not guaranteed or endorsed by the publisher.
